# Ciguatoxin reduces regenerative capacity of axotomized peripheral neurons and delays functional recovery in pre-exposed mice after peripheral nerve injury

**DOI:** 10.1038/srep26809

**Published:** 2016-05-27

**Authors:** Ngan Pan Bennett Au, Gajendra Kumar, Pallavi Asthana, Chung Tin, Yim Ling Mak, Leo Lai Chan, Paul Kwan Sing Lam, Chi Him Eddie Ma

**Affiliations:** 1 Department of Biomedical Sciences, City University of Hong Kong, Tat Chee Avenue, Hong Kong; 2 Department of Mechanical and Biomedical Engineering, City University of Hong Kong, Tat Chee Avenue, Hong Kong; 3 Centre for Biosystems, Neuroscience, and Nanotechnology, City University of Hong Kong, Tat Chee Avenue, Hong Kong; 4 State Key Laboratory in Marine Pollution, City University of Hong Kong, Tat Chee Avenue, Hong Kong; 5 Shenzhen Key Laboratory for the Sustainable Use of Marine Biodiversity, Research Centre for the Oceans and Human Health, City University of Hong Kong Shenzhen Research Institute, Shenzhen, China; 6 Department of Biology and Chemistry, City University of Hong Kong, Tat Chee Avenue, Hong Kong

## Abstract

Ciguatera fish poisoning (CFP) results from consumption of tropical reef fish containing ciguatoxins (CTXs). Pacific (P)-CTX-1 is among the most potent known CTXs and the predominant source of CFP in the endemic region responsible for the majority of neurological symptoms in patients. Chronic and persistent neurological symptoms occur in some CFP patients, which often result in incomplete functional recovery for years. However, the direct effects of exposure to CTXs remain largely unknown. In present study, we exposed mice to CTX purified from ciguatera fish sourced from the Pacific region. P-CTX-1 was detected in peripheral nerves within hours and persisted for two months after exposure. P-CTX-1 inhibited axonal regrowth from axotomized peripheral neurons in culture. P-CTX-1 exposure reduced motor function in mice within the first two weeks of exposure before returning to baseline levels. These pre-exposed animals exhibited delayed sensory and motor functional recovery, and irreversible motor deficits after peripheral nerve injury in which formation of functional synapses was impaired. These findings are consistent with reduced muscle function, as assessed by electromyography recordings. Our study provides strong evidence that the persistence of P-CTX-1 in peripheral nerves reduces the intrinsic growth capacity of peripheral neurons, resulting in delayed functional recovery after injury.

Ciguatera fish poisoning (CFP) is the most prevalent type of human food poisoning resulting from the ingestion of marine fish containing ciguatoxins (CTXs); CFP affects more than 50,000 people worldwide annually, despite of the fact that under-diagnosis and under-reporting are common^1^,^2^,^3^. The consumption of CTX-contaminated fish poses a significant health risk, even at a concentration as low as 0.1 ppb^4^,^5^. CTX-contaminated fish can be found in endemic areas to particular regions of the Pacific Ocean, Indian Ocean and Caribbean regions. Pacific CTX-1 (P-CTX-1) is among the most potent of the known CTXs and is the predominant source of CFP in the region of Pacific Ocean, accounting for the majority of neurological symptoms in patients^6^. Within hours of ingesting CTX-contaminated fish, patients can exhibit a diverse range of neurological and gastrointestinal symptoms, including muscle weakness, paralysis, limb paraesthesia, diarrhoea, abdominal pain and nausea, with gastrointestinal symptoms generally appearing first^3^,^7^,^8^. The gastrointestinal symptoms usually last for approximately one week; however, severe neurological manifestations can last for months or years in 20% of patients, and approximately 2–5% of patients exhibit incomplete recovery years after exposure^3^,^9^. Relapsing ciguatera has been documented several years after recovery from the first onset of the syndrome, and the consumption of CTX-contaminated fish further worsens these neurological symptoms^2^,^3^,^7^,^9^. Therefore, we reasoned that the persistence of CTXs in the body contributes to these chronic neurological manifestations and relapsing ciguatera observed in patients with CFP. However, the chronicity and persistence of CTXs in the nervous system are poorly understood.

Several lines of evidence suggest that CTXs affect the peripheral nervous system (PNS). Specifically, CTXs can bind to sodium channel receptors on both somatic and autonomic nerves and activate voltage-sensitive sodium channels (VSSCs) in sciatic nerves, causing the influx of sodium into axons^8^,^10^. P-CTX-1 increases the excitability of cultured sensory neurons (dorsal root ganglions, DRGs) by modulating sodium channel gating. Importantly, subsequent washout with a CTX-free solution did not abolish the activity of P-CTX-1, suggesting persistent and irreversible effects for P-CTX-1 in axotomized peripheral neurons^11^. A significant reduction in myelin fibre density, axon demyelination, and axonal degeneration with oedema were observed in patients with CFP who developed peripheral neuropathy^8^,^12^,^13^ and displayed incomplete functional recovery^7^,^8^,^13^,^14^. However, the reasons for these prolonged peripheral neurological disturbances remain largely unknown. P-CTX-1 was reported to evoke acute peripheral sensory and locomotion disturbances within hours in treated mice, with symptoms including a hypothermic response, cold allodynia, chronic fatigue, muscle weakness, and decreased nerve conduction velocity^15^,^16^. In addition, P-CTX-1 elicited the rapid onset of cold allodynia within hours after exposure by targeting myelinated and unmyelinated DRG neurons via TRPA1-dependent calcium influx^15^. Epidemiological studies identified strong peripheral and neurological associations with CFP cases, consistent with the findings from experimental animal studies.

Unlike in the central nervous system (CNS), where regeneration after axonal injury is poor, neurons in the PNS can successfully regenerate their axons, although the rate of axonal growth is slow (1–2 mm/day in humans and rodents)^17^,^18^. DRG neurons provide a useful model for studying peripheral regenerative mechanisms, and they are widely used to assess cues that promote or inhibit axonal outgrowth during peripheral nerve regeneration^17^,^18^,^19^,^20^. These somatosensory neurons are unipolar, with an axon that splits in two, sending one branch to the peripheral target (muscle) through the peripheral nerve (sciatic nerve) and the other to the brainstem for processing. In animal models of peripheral nerve crush injury, the peripheral nerve can regenerate successfully and regain its full sensory and motor function within approximately one month of a crush injury^18^. Nevertheless, the absence of an effective blood-nerve barrier in the sensory neurons and nerves of the PNS are at increased risk as compared with the CNS, which is relatively well protected by the blood-brain barrier.

Here, we show that P-CTX-1, which was purified from ciguatera fish sourced from the Pacific Ocean^6^,^21^, can be detected in the sciatic nerve and other major tissue organs within hours and remains up to two months after exposure. P-CTX-1 inhibits the regenerative capacity of axotomized primary peripheral neurons in a sodium-dependent manner *in vitro*. Repeated exposure (twice) to P-CTX-1 reduces motor strength in mice within the first two weeks before returning to baseline levels. These pre-exposed baseline animals show a substantial delay in sensory function recovery and irreversible motor function deficits after sciatic nerve crush injury. Additional histological and electrophysiological studies revealed that the motor deficits were largely due to decreased functional synapse reinnervation at the motor end plate in the distal plantar muscle. These findings provide further evidence for the persistence and chronicity of P-CTX-1 on PNS regeneration.

## Results

### P-CTX-1 chronically persists in the peripheral nervous system

**Figure 1:**
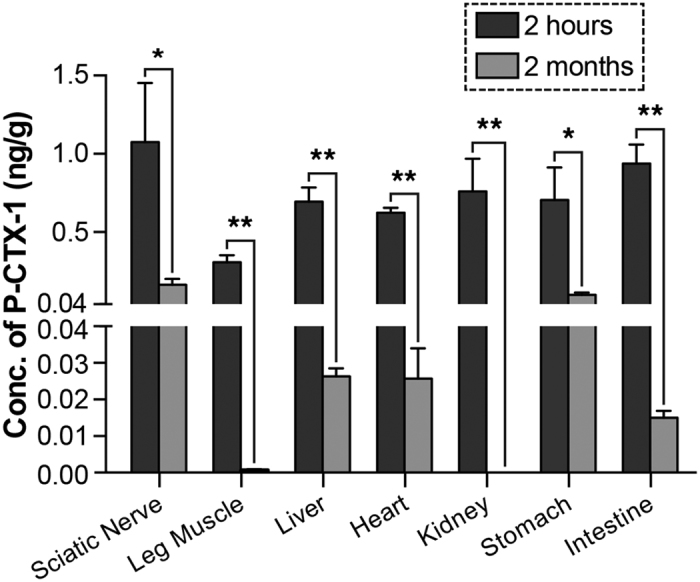
P-CTX-1 persists in the nervous system *in vivo* after repeated exposure. Adult mice (n = 3) were intraperitoneally injected with 0.26 ng/g P-CTX-1 on days 0 and 3, and their tissues were harvested two hours or two months after the last injection. P-CTX-1 levels were then estimated using the mouse neuroblastoma assay. P-CTX-1 was detected in the major organs shortly after the second injection; levels were significantly reduced two months after the last injection in most tissues but remained high in the sciatic nerve (n = 3 mice per group; mean ± SEM; *P < 0.05, **P < 0.01, one-way ANOVA, followed by Bonferroni’s post hoc test).

Unlike the gastrointestinal symptoms of CFP, neurological symptoms take longer to develop and are apparent several days after the toxin affects the PNS^22^. In severe cases, patients develop peripheral neuropathology targeting the PNS, with the characteristic features of Guillain-Barré syndrome (GBS)^23^. Several studies in both patients and animals have shown a direct correlation between CTX levels in fish and the severity of neurological symptoms^24^,^25^,^26^, suggesting a potential link between CTX levels in the blood of patients and CFP severity^26^. Due to the lipid-soluble polyether chemical nature of CTXs, it is not surprising that P-CTX-1 can be detected in the blood, excreta, muscles, and brains of mice and rats up to 96 hours after intraperitoneal (i.p.) injections^16^,^27^; however, studies on the accumulation of P-CTX-1 over long periods of time are scarce. Therefore, we hypothesized that the persistence of CTXs in peripheral nerves contributes to the chronic neurological manifestations in the PNS. To investigate the persistence of P-CTX-1 in the PNS and major organs, sub-lethal doses of P-CTX-1 (0.26 ng/g) were intraperitoneally administered to naïve mice twice (days 0 and 3)^16^. Intact sciatic nerves, leg muscles, and other major organs, including the liver, heart, kidney, intestine and stomach, were harvested directly from pre-exposed mice two hours or two months after the last injection. The concentrations of P-CTX-1 in various tissues were then quantified using a cell-based mouse neuroblastoma assay (MNA)^6^,^21^. Two hours after the second administration, the highest levels of P-CTX-1 were detected in the sciatic nerve (>1 ng/g), followed by the major organs, including the intestine, kidney, liver, stomach and heart (0.5–1 ng/g). P-CTX-1 levels were significantly reduced two months after the last administration in various organs, although they remained at the highest levels in the sciatic nerves relative to the other tissues (Fig. 1; Supplementary Table S1). These results indicate that P-CTX-1 accumulates in PNS tissues, which could contribute to the persistent peripheral neurological effects observed in patients with CFP^8^,^9^, even though sciatic nerves are protected by the blood-nerve barrier.

### P-CTX-1 reduces axonal regrowth in cultured DRG neurons

**Figure 2:**
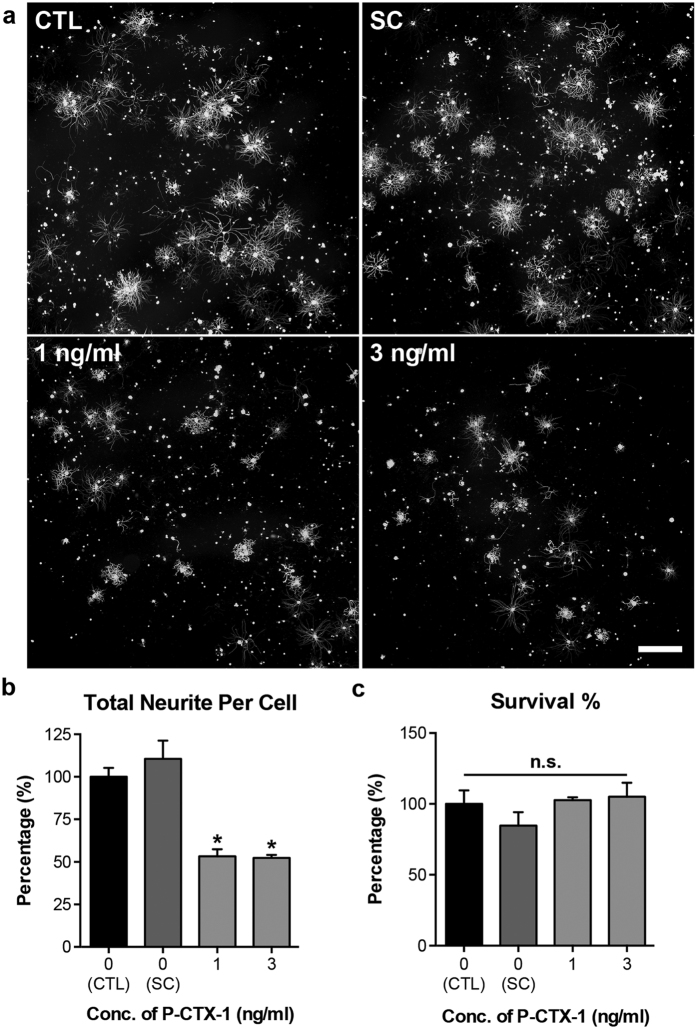
P-CTX-1 exerts a strong inhibitory effect on the regenerative capacity of axotomized DRG neurons. **(a)** Representative photomicrographs of DRG neurons immunolabelled with antibodies against anti-β-tubulin III, showing dramatically shorter and less extensive neurites in the presence of P-CTX-1. Scale bar: 500 μm. **(b)** At 1 and 3 ng/ml, P-CTX-1 reduced neurite length in axotomized primary DRG neurons. There was no detectable inhibitory effect of 0.15% methanol [solvent control (SC) of P-CTX-1] on the regrowth of the DRG neurites (mean ± SEM of triplicates; *P < 0.05, one-way ANOVA, followed by Bonferroni’s post hoc test). **(c)** The relative percentage of viable DRG neurons grown in solvent control (SC, 0.15% methanol), and P-CTX-1-treated culture was comparable to those grown in culture medium (CTL) (mean ± SEM of triplicates; one-way ANOVA, followed by Bonferroni’s post hoc test). n.s., not significant.

**Figure 3:**
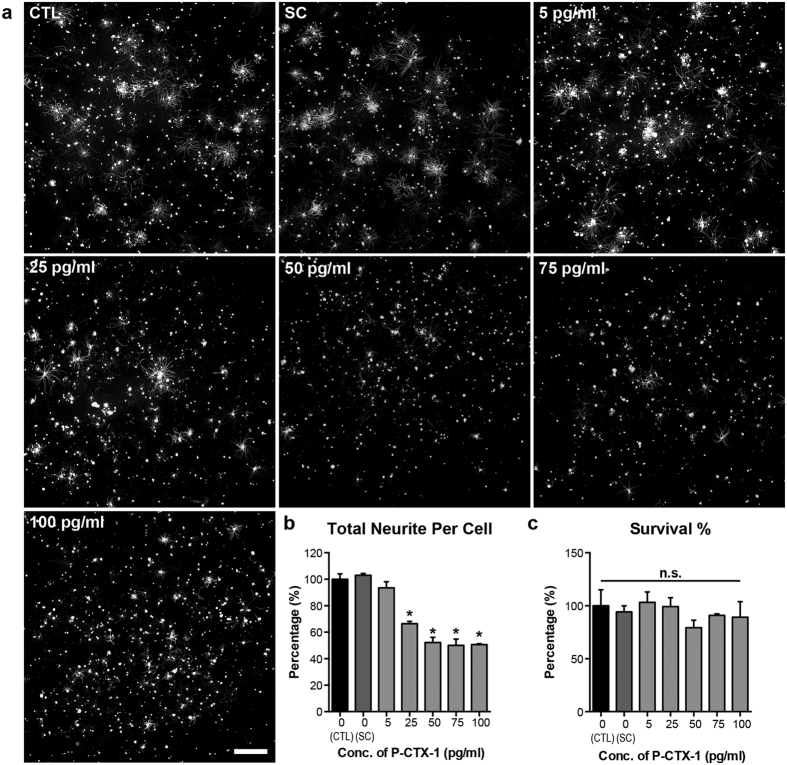
Excessive sodium ion influx enhances the inhibitory effects of P-CTX-1 on DRG neurons *in vitro*. **(a)** Representative photomicrographs of DRG neurons immunolabelled with antibodies against anti-β-tubulin III. At concentrations ranging from 25 to 200 pg/ml, P-CTX-1 greatly reduced neurite extension in DRG neurons in the presence of 50 μM ouabain and 5 μM veratridine (sodium channel activators that increase sodium influx). Scale bar: 500 μm. **(b)** The total length of neurites in the DRG neurons was significantly reduced in all of the P-CTX-1-treated groups, with the exception of the 5 pg/ml group (mean ± SEM of triplicates; *P < 0.05, one-way ANOVA, followed by Bonferroni’s post hoc test). **(c)** No detectable effects on DRG neuron survival were observed in the P-CTX-1-treated groups or the controls (CTL). All treatment conditions, including the CTL and solvent control (SC, 0.13% methanol), contained 5 μM veratridine and 50 μM ouabain. n.s., not significant.

Peripheral neurological disturbances are commonly observed in patients with CFP, in which nerve damage have been well documented^8^,^12^,^13^; however, the direct effects of these toxins on nerves remains largely uncharacterized. These reports prompted us to investigate the direct effects of P-CTX-1 on peripheral DRG neurons. Successful axon regeneration depends on the intrinsic regenerative capacity of the neurons following lesion^18^. Therefore, we examined the regenerative capacity of axotomized primary DRG neurons following acute exposure to P-CTX-1. P-CTX-1 significantly inhibited axonal outgrowth from primary DRG neurons (Fig. 2a,b) by approximately 50% at concentrations of 1 and 3 ng/ml, without exhibiting any effects on neuronal survival (Fig. 2c). P-CTX-1 is a sodium channel activator, and it induces excessive sodium ion influx^2^. Therefore, we next examined whether the inhibitory effects of P-CTX-1 on neurite outgrowth are sensitive to sodium levels by increasing sodium influx using ouabain (which blocks sodium efflux via Na^+^/K^+^ ATPase) and veratridine (a sodium channel activator that binds to site 2 of VSSCs)^6^. The inhibitory effects of low-dose P-CTX-1 treatment (25 pg/ml) were greatly enhanced in the presence of excess sodium ions (Fig. 3a,b), without affecting the survival of the DRG neurons (Fig. 3c).

**Figure 4:**
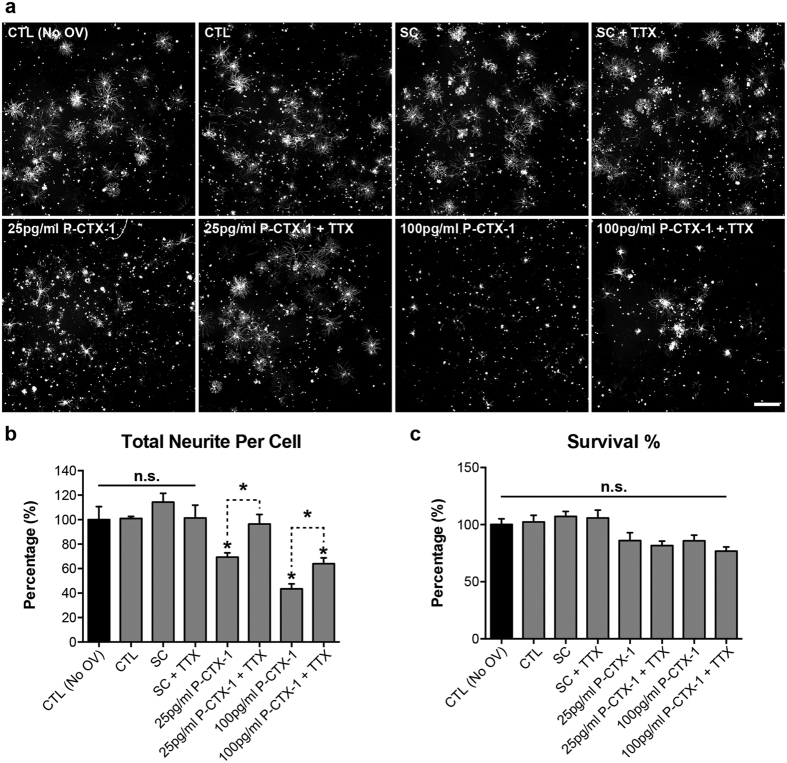
Blockade of excess sodium ion influx abolishes the growth inhibitory effects of P-CTX-1 on DRG neurons *in vitro*. **(a)** Representative photomicrographs of DRG neurons immunolabelled with antibodies against anti-β-tubulin III. DRG neurons treated with 0.3 μM TTX were able to extend their neurites to lengths comparable to the solvent controls (SC, 0.13% methanol) in the presence of 25 pg/ml P-CTX-1. TTX-treated DRG neurons exhibited significantly longer neurites in the presence of 100 pg/ml P-CTX-1 than those treated with 100 pg/ml P-CTX-1 alone. Scale bar: 500 µm. **(b)** Blockade of sodium influx with TTX significantly enhanced neurite extension in DRG neurons treated with 25 and 100 pg/ml P-CTX-1. **(c)** Neither P-CTX-1 nor TTX affected the survival of DRG neurons at any of the tested concentrations. The values represented the mean ± SEM values of triplicates; *P < 0.05, one-way ANOVA, followed by Bonferroni’s post hoc test. All treatment conditions, except for CTL (medium only), contained 50 μM ouabain (O) and 5 μM veratridine (V). There was no significant difference in neurite outgrowth between DRG neurons cultured with or without O/V. CTL, control; OV, 50 μM ouabain and 5 μM veratridine; TTX, 0.3 μM tetrodotoxin; n.s., not significant.

To further test our hypothesis that P-CTX-1-induced inhibition of axonal growth is dependent on sodium influx, we applied 0.3 μM tetrodotoxin (TTX) to the neurons one hour prior to treatment with 25 or 100 pg/ml (the highest dose tested) P-CTX-1. TTX is a well-known sodium channel blocker that effectively blocks the excessive influx of sodium ions at a concentration of 0.3 μM^28^. TTX completely restored the regenerative capacity of the DRG neurons that were treated with 25 pg/ml P-CTX-1 and partially restored neurite outgrowth in DRG neurons that were treated the highest dose (100 pg/ml) of P-CTX-1 (Fig. 4a,b), without inducing cytotoxicity (Fig. 4c). Therefore, we conclude that P-CTX-1 inhibits neurite outgrowth from DRG neurons in a sodium-dependent manner. Taken together, these findings formed the basis of our major hypothesis that a reduction in the intrinsic growth capacity of adult neuron could have long-term impacts on regenerative capacity in animals.

### Repeated exposure to P-CTX-1 impairs motor coordination and strength, and delays functional recovery following peripheral nerve injury *in vivo*

**Figure 5:**
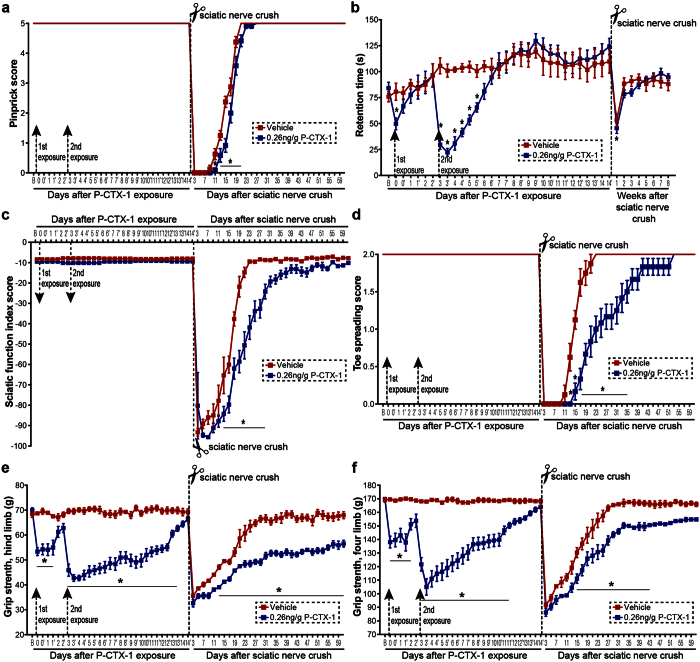
Pre-exposure to P-CTX-1 impairs sensory and motor function recovery after sciatic nerve crush. All behavioural tests were performed in two sessions set eight hours apart (morning session −1, evening session −1’) during the first two weeks, and the test results had returned to the baseline values before we performed crush injuries. **(a)** The P-CTX-1 (0.26 ng/g)-treated mice showed a marked delay in sensory recovery after injury, as assessed by the pinprick reflex, and full recovery was also delayed. **(b)** Repeated exposure to P-CTX-1 significantly reduced retention time on the rod compared with controls. Crush injury further reduced the retention time. **(c)** The SFI values remained unchanged after repeated exposure to P-CTX-1, and the recovery of the P-CTX-1-treated group lagged behind that of controls over the time course. **(d)** The P-CTX-1-treated mice had lower toe spreading scores from days 13 to 35, and full recovery was delayed by 30 days. **(e,f)** Grip strength of hind limbs (**e**) and four limbs (**f**) was significantly reduced after the first and second P-CTX-1 treatments. The hind limb grip strength of these mice never returned to the baseline value after crush injury compared with the controls (n = 10–12 mice per group; mean ± SEM; *P < 0.05, two-way repeated measures ANOVA, followed by Bonferroni’s post hoc test). B, baseline.

We first tested for acute neurological effects of P-CTX-1 on sensory and motor function using an extensive battery of behavioural tests, which were performed in a sequential manner^18^. We then examined the chronic effects of P-CTX-1 in an animal model of sciatic nerve crush injury in which adult mice require approximately one month to regain full functional recovery after crush^18^. Any reduction in the regenerative capacity of peripheral neurons results in partial functional recovery of the injured mice after exposure to P-CTX-1, as reflected by the outcomes of the behavioural tests. The sciatic nerve crushes were performed on day 14, at which time all detectable behavioural abnormalities in the mice had returned to normal after the second exposure (Fig. 5). Analogous to patients with CFP, exposure of adult mice to P-CTX-1 caused diarrhoea during the first week and body weight loss, which returned to normal levels two weeks after the first exposure (Supplementary Fig. S1). Therefore, the potency of P-CTX-1 was confirmed in this study. Sensory function was assessed by applying gentle pinprick stimuli to the lateral plantar surface of the paw. We did not observe sensory deficits (pinprick assay) after the first two exposures; however, the return of sensory function with respect to axonal growth rate was significantly reduced in the pre-exposed animals between days 11 and 21 after sciatic nerve crush. Moreover, the mice pre-exposed to P-CTX-1 took two and four days longer to show the initial and full responses, respectively, compared with the vehicle controls (Fig. 5a). The delay in sensory functional recovery agrees well with our *in vitro* data, showing that P-CTX-1 reduced the regenerative capacity of injured peripheral neurons.

Motor coordination (rotarod test) was significantly affected, and trembling was observed during the first week after exposure to P-CTX-1 due to hypothermia^15^. Following sciatic nerve crush, motor coordination in both groups was significantly reduced during the first week after injury, and became largely unaffected in the following weeks which were indistinguishable from baseline values (Fig. 5b). The sciatic function index (SFI) and toe spreading remained unchanged after exposure to P-CTX-1 (Fig. 5c,d), since both tests are commonly used to assess motor function recovery after sciatic nerve injury. By contrast, we observed that gait movement, as assessed by SFI, was strongly impaired from day 13 to 27 after injury (Fig. 5c). To further evaluate motor function recovery, we performed the toe spreading test and observed a 30-day delay in reaching full recovery (score 2) (Fig. 5d). Motor function was quantitatively assessed using a grip strength meter, and we found that grip strength was reduced from 170 g to 105 g, which slowly returned to baseline levels within two weeks (Fig. 5e,f). However, the grip strength of the P-CTX-1-exposed animals failed to return to baseline levels, even by two months post-injury (Fig. 5e,f), clearly demonstrating that chronic exposure to P-CTX-1 exhibited adverse effects on functional recovery.

### P-CTX-1 reduces axon density and motor end plate reinnervation in pre-exposed animals following sciatic nerve injury

**Figure 6:**
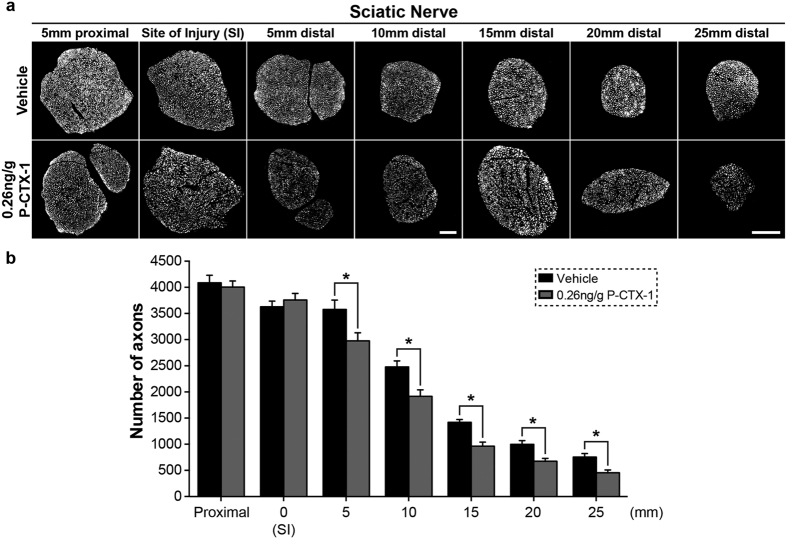
P-CTX-1 pre-exposure reduces the number of regenerating axons. **(a)** Four micron-thick transverse sections of the sciatic nerve were immunostained with an anti-neurofilament NF200 antibody, and the total number of axons was quantified two months after sciatic nerve crush. Scale bar: 100 μm. **(b)** Quantification of the total number of regenerating axons showed a significant reduction in the number of axons 5–25 mm distal to the site of injury (SI) on the ipsilateral side compared with the vehicle controls. (n = 3–4 mice per group and 5–8 sections from each mouse; mean ± SEM; *P < 0.05, Student’s *t*-test).

Motor function recovery depends on the successful nerve terminal and muscle reinnervations, and eventually the formation of a functional neuromuscular junction (NMJ) at the motor end plate^18^. We next tested whether the irreversible motor strength deficits observed two months after P-CTX-1 exposure were associated with axon loss and a lack of motor end plate reinnervation in animals. We first quantified the number of neurofilament-200 (NF200)-positive axons in the sciatic nerve 5–25 mm distal to the crush site. The number of axons was significantly reduced in the P-CTX-1-treated mice on the ipsilateral side of the injury site (injured nerves) two months after crush (Fig. 6), and the axon density remain largely unchanged on the contralateral side (uninjured nerves) (Supplementary Fig. S2). Our data suggests that P-CTX-1 does not affect the intact nerves but mainly targeting the regenerating axons.

**Figure 7:**
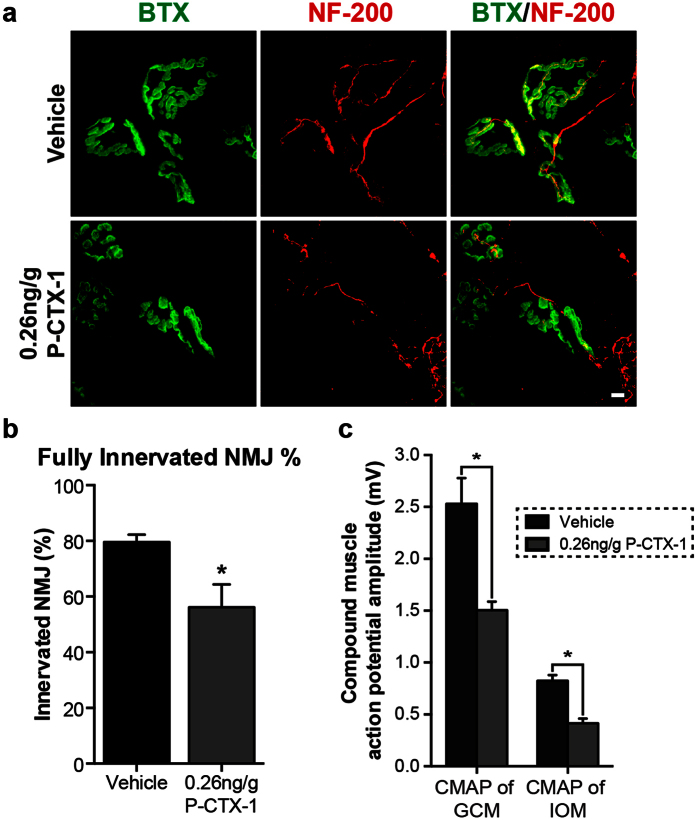
Chronic exposure to P-CTX-1 impairs the formation of functional synapses after sciatic nerve injury. **(a)** α-bungarotoxin (BTX; green) and neurofilament (NF200; red) co-immunostaining showed a relative reduction in NMJ reinnervation in the ipsilateral plantar muscle of the P-CTX-1-treated mice two months after injury. Scale bar: 5 μm. **(b)** The graph shows the quantification of the number of innervated NMJs (n = 6–8 mice per group; mean ± SEM; *P < 0.05, Student’s *t*-test). **(c)** The CMAP amplitudes of the ipsilateral GCM and IOM were significantly decreased in the P-CTX-1-treated mice two months after injury, consistent with our behavioural and NMJ data. (n = 5 mice per group; mean ± SEM; *P < 0.05, one-way ANOVA, followed by Bonferroni’s post hoc test).

We further investigated whether the motor function deficit was solely due to the lack of axon regrowth into the distal muscle or whether there was also a loss of motor end plate reinnervation in the existing axons. NMJ analysis revealed a greater than 20% reduction in NMJ reinnervation in the interosseous (plantar) muscle in P-CTX-1-exposed mice compared with vehicle controls (Fig. 7a,b). These results agree closely with the outcomes of the motor function behavioural tests.

We next performed *in vivo* electromyography (EMG) recordings in anaesthetized mice during the course of recovery to assess synapse formation following injury. The EMG recordings allowed us to detect functional neuromuscular synaptic transmission at the target distal muscles, which is commonly used as a method to quantify motor functional recovery, as well as to validate the results of our motor function behavioural tests. The compound muscle action potentials (CMAPs) of the gastrocnemius and interosseous muscles were evaluated two months after sciatic nerve injury. Compound muscle action potentials amplitude was significantly reduced in the muscles proximal (gastrocnemius) and distal (interosseous) to the injured sites (Fig. 7c; Supplementary Fig. S3). CMAP studies provide an important electrophysiology parameter that measures the conduction velocity of peripheral nerves, and these results further confirmed our behavioural and histological data. Taken together, we conclude that pre-exposure to P-CTX-1 impairs peripheral axon regeneration and functional recovery after nerve injury.

## Discussion

CTXs are among the most potent of known naturally occurring neurotoxins, and they are heat-stable, colourless, odourless, and cannot be easily detected or destroyed, even by prolonged refrigeration or cooking. CFP is one of the most challenging foodborne illnesses in clinical medicine; however, the chronicity and persistence of CTXs in the nervous system are poorly understood. CTXs originate from gambiertoxins (GTXs) synthesized by dinoflagellates (*Gambierdiscus toxicus*)^29^. Herbivorous fish ingest GTX-containing dinoflagellates, bioaccumulating these chemicals, which in turn are progressively concentrated in carnivorous coral reef fish as one moves up the food chain. Finally, GTXs are biotransformed into CTXs, ultimately affecting apex carnivores – humans.

The gastrointestinal symptoms (e.g., diarrhoea) of CFP generally develop within 6–24 hours and spontaneously resolve within 1–2 weeks. However, the neurological symptoms of CFP usually take longer to develop and resolve after disease onset. Acute neurological symptoms resolve within several weeks, although chronic symptoms, such as fatigue, weakness and depression, can persist for months or even years^8^. Gastrointestinal symptoms, such as diarrhoea, were observed in the P-CTX-1-treated mice and lasted for two days after administration, which then subsided with a significant decrease in body weight, consistent with other reports^16^,^30^. Body weight decreased by 27.5% after the second P-CTX-1 exposure and then returned to normal baseline levels within two weeks, clearly demonstrating the potency of the P-CTX-1 used in this study.

We detected exceptionally high levels of P-CTX-1 in lipid-rich neuronal tissues (e.g., the sciatic nerve) two days after the second exposure. Additionally, elimination of P-CTX-1 from the sciatic nerves was seemingly inefficient as we were still able to detect a significant amount of the toxin long after exposure. As P-CTX-1 is lipophilic in nature^8^, it is not surprising that P-CTX-1 has a greater tendency to accumulate in lipid-rich tissues such as the brain^27^ and nerves. Therefore, we reasoned that adverse effects following injury, such as motor function deficits, axonal loss, and impaired axon regeneration, were likely to be secondary effects caused by the persistence of P-CTX-1 in the nervous system.

Several lines of evidence suggest that chronic exposure to P-CTX-1 associated with severe neurological manifestations in the PNS of some patients^8^,^13^,^22^. Severe neurological symptoms, including muscle weakness (particularly in the muscles innervated by the cranial nerves), paresis of the left facial nerves, a marked decrease in the tendon reflex, and loss of ankle and abdominal reflexes, can develop within one week, shortly after the gastrointestinal symptoms appear. EMG recordings revealed the development of peripheral neuropathy in the lower limbs of patients. Sural nerves from biopsy samples show a loss of myelinated fibres, oedema, and axonal degeneration, with no detectable neurological manifestations nine months after CFP onset^13^. Therefore, we hypothesized that the neurotoxicity of P-CTX-1, similar to other peripheral neuropathologies such as diabetic neuropathology and chemotherapy-induced peripheral neuropathy, primarily affects the PNS. We show that P-CTX-1 specifically targets the PNS as the highest levels of P-CTX-1 accumulated in the sciatic nerve two months after exposure. A previous study demonstrated that a relatively low level of P-CTX-1 was detected in rat brains 96 hours after a single exposure to P-CTX-1^27^. These studies provide strong evidence that prolonged exposure to P-CTX-1 leads to progressive axon loss. The mechanism responsible for this loss is unclear, but results suggest that P-CTX-1 might have an effect on axonal regeneration in peripheral neurons. This hypothesis agrees well with our data, which clearly show that P-CTX-1 exerts its inhibitory effects on axonal regrowth from axotomized DRG neurons *in vitro* and *in vivo*, without a significant effect on survival of the cultured peripheral neurons. Recent findings indicate that axon damage and a reduction in the regenerative capacity of neurons are linked to the dysregulation of sodium channel activity^28^,^31^. Excess sodium ion influx and aberrant ionic homeostasis of sodium/potassium ions in the DRG neurons reduce neurite outgrowth *in vitro*^28^. We found that excessive sodium ion influx induced by ouabain and veratridine enhanced the inhibitory effects of a low concentration of P-CTX-1. This sodium-dependent, P-CTX-1-mediated inhibitory effect was further validated using the sodium channel blocker TTX. Treatment with TTX was able to rescue neurite outgrowth from DRG neurons following inhibition with a relatively low dose of P-CTX-1 (25 pg/ml), although axonal regrowth was only partially restored following treatment with a high concentration of P-CTX-1 (100 pg/ml). It is possible that P-CTX-1 can differentially modulate the activity of both TTX-sensitive and TTX-resistant sodium channels^11^. TTX has been shown to block only a subset of sodium channels (i.e., TTX-sensitive sodium channels)^32^. Therefore, it is not surprising that P-CTX-1 exerts its growth-inhibitory effect on DRG neurons partly via the activation of TTX-resistant sodium channels, which cannot be blocked by TTX. These data are in agreement with previous studies showing that P-CTX-1 can directly bind to VSSCs in DRG neurons and cause an excessive influx of sodium ions^11^,^33^, activating sodium channels and inducing swelling at the nodes of Ranvier in myelinated axons in frogs^10^,^34^. Consistent with our findings, the adverse effects of P-CTX-1 on myelinated axons are attenuated after pre-treatment with the sodium channel blocker TTX^11^,^33^.

Patients with CFP exhibiting marked neurological abnormalities manifested as moderate or severe motor deficits have been reported^9^,^14^,^30^,^35^,^36^. The restoration of motor functions following peripheral nerve injury requires not only the successful regeneration of injured axons but also their reinnervation to their original muscle targets and forming a functional NMJ^18^. Degeneration of the distal nerve stump usually occurs (Wallerian degeneration) following peripheral nerve injury, although damaged proximal axons can successfully regenerate and reinnervate their distal targets; thus, functional recovery is expected^37^,^38^. Exposure to chemotherapeutic drugs or neurotoxins inhibits axonal regeneration, and in such cases, proximal peripheral axons cannot reinnervate their targets after peripheral nerve injury^20^,^39^,^40^. A delay in muscle reinnervation is associated with muscle atrophy, alterations to muscle electrophysiology, and ultimately motor function deficits^18^,^41^,^42^,^43^. In the current study, we detected P-CTX-1 in the sciatic nerve and showed a reduction in the number of nerve terminals and muscle reinnervations as well as a decrease in the number of functional NMJs; these effects were accompanied by a reduction in CMAP levels in P-CTX-1 pre-exposed mice, consistent with the persistence of P-CTX-1 in the PNS. The binding of P-CTX-1 to VSSCs was irreversible in DRG neurons^11^; therefore, we speculate that the persistent effects of the toxin are correlated with the long-term enhancement of neuronal excitability caused by P-CTX-1-induced excessive sodium ion influx. All of our findings agree with the data from our behavioural assessments showing that muscle strength (measured by grip strength) in the pre-exposed mice does not fully recover two months after peripheral nerve injury.

Taken together, our studies show that the accumulation of P-CTX-1 in the nervous system can be detrimental to nerve regeneration and functional recovery following injury. Apart from the gastrointestinal symptoms, the onset of neurological symptoms is slower and may go unnoticed for several years as P-CTXs can persist in the PNS for long periods of time. Our findings suggest that the accumulation of P-CTX-1 in the PNS, affecting functional NMJ formation and motor function recovery after peripheral nerve injury.

## Methods

### Animals

All experiments and euthanasia were conducted in compliance with the IACUC guidelines. Adult male mice (C57BL/6, 8–12 weeks) were used for all *in vitro* and *in vivo* experiments. The animals were provided with food and water *ad libitum*, with a 12 h light-dark cycle. Animal experimental protocols were approved by the Animal Research Ethics Sub-Committee in City University of Hong Kong and Department of Health, HKSAR.

### P-CTX-1 purification and administration

Based on the P-CTX-1 standard kindly provided by Professor Richard Lewis (University of Queensland), 40 μg of P-CTX-1 was isolated and purified from viscera of moray eels (Gymnothorax spp.) (5.6 kg) collected from the Republic of Kiribati as previously described^6^,^21^. Briefly, viscera was cooked at 70 °C and then extracted with acetone. The acetone extract was filtered and dried using a rotary evaporator. The resulting acetone extract was partitioned between hexane and 90% aqueous methanol, and the residue in methanolic phase was further partitioned between 25% ethanol and diethyl ether. The residue in diethyl ether layer was subjected to different types of column chromatography with packing materials including Florisil, Sephadex LH20, and HW 40S. Further purification was carried out using Hamilton PRP-1 (150 × 4.1 mm i.d., 5 μm) and Phenomenex Luna C18 (2) (250 × 2.0 mm i.d., μm) columns. Fractions containing P-CTX-1 were determined by comparing the retention times with standards using high-performance liquid chromatography–tandem mass spectrometry (HPLC-MS/MS) (AB SCIEX QTRAP^®^ 5500). Purified P-CTX-1 was reapplied to HPLC columns and eluted with solvents of different polarities to confirm homogeneity. Purity of the authentic P-CTX-1 standard was over 95% by normalization of peak areas detected by HPLC-diode array detectors.

P-CTX-1 was dissolved in 1% phosphate buffered saline (PBS)-Tween 60. Mice were weighed and randomly divided into vehicle control and P-CTX-1-treated groups. A sub-lethal dosage of purified P-CTX-1 (0.26 ng/g body weight) or vehicle control (PBS solution with 1% Tween 60) was administered intraperitoneally to adult C57BL/6 mice (8–12 weeks old) on day 0 and 3. Body weight was monitored throughout the behavioral study.

### P-CTX-1 tissue distribution determined by mouse neuroblastoma assay

We harvested all intact organs and tissues from terminally anesthetized mice (followed by cervical dislocation) 2 hours or 2 months after the second administration of P-CTX-1 via intraperitoneal injection. All organs and tissues including sciatic nerve, leg muscle, liver, heart, kidney, stomach and intestine were freshly dissected from intact P-CTX-1-treated mice (n = 3) for P-CTX-1 levels analysis. All the harvested tissues were immediately transferred to −80 °C for storage. P-CTX-1 was extracted from the harvested tissues and the P-CTX-1 concentration was determined by using MNA^6^,^21^. Neuro-2a cells were seeded at a density of 2.5 × 10^5^ cells per well in 96-well plates supplemented with RPMI-1640/10% fetal bovine serum. After 24 hours, medium was renewed with medium containing 50 μM ouabain (O) and 5 μM veratridine (V) (Sigma-Aldrich). Cells will be dosed with P-CTX-1 standards at seven concentrations range from 0.97 pg/ml to 62.5 pg/ml in five replicates. Tissue extracts was tested in triplicates and cell viability was measured by MTT assay as described previously^6^,^21^. LC_50_ values (0.103 ± 0.0173 ng/ml) were determined from the dose-response curve and the ciguatera toxicity of samples was determined from the standard curve (Supplementary Fig. S4). Data was obtained from at least three separate experiments.

### Primary dissociated DRG cultures

Dissociated DRG cultures were prepared from adult C57BL/6 mice as previously described^17^,^18^,^20^. Briefly, DRGs were dissected out and mildly digested by collagenase and dispase II (Roche Diagnostics). Cells were trypsinized and mechanically dissociated using three flame-polished Pasteur pipettes with different diameters. Two thousand DRG neurons were plated onto a poly-D-lysine and laminin (Sigma-Aldrich)-coated 8-well chamber slides (Millipore) containing Neurobasal medium (Gibco) supplemented with B27, penicillin-streptomycin, 200 mM L-glutamine, 50 ng/ml NGF (Gibco), 2 ng/ml GDNF and 10 μM Ara-C (Sigma-Aldrich). The cultures were allowed to grow with or without 50 μM O and 5 μM V as indicated. To block the voltage-gated sodium channels, TTX (Alomone Labs) was added to the cultures at a final concentration of 0.3 μM. P-CTX-1 was first dissolved in 20% methanol/PBS solution and added to the cultures at indicated concentrations. The final concentration of methanol was at 0.15% (without O and V) in Fig. 2 or 0.13% (with O and V) in Figs 3 and 4, as solvent control (SC). There was no statistical difference in the neurite outgrowth between DRG neurons cultured with or without O/V (Fig. 4).

### Neurite outgrowth and survival assay

After 17 hours of incubation, the cultures were fixed with 4% paraformaldehyde (PFA), blocked with 0.5% bovine albumin/0.1% Triton X-100 (Sigma-Aldrich) in PBS and incubated with anti-β-tubulin III (mouse monoclonal, 1:800, Sigma-Aldrich) for overnight at 4 °C. The cultures were washed with PBS and incubated with Alexa Fluor 488-conjugated secondary antibody (1:300, Molecular Probes) for neurite outgrowth assay. 30 non-overlapping images were taken at 10× magnifications using a motorized fluorescence microscope (Nikon Eclipse 90i) for analysis. Total neurite length of DRG neurons of individual neurons from each condition was measured by automated WIS-NeuroMath software (Weizmann Institute of Science)^17^,^18^. The total neurite length of the neurons was averaged from at least 250 neurons per condition. Neurons with phase-bright cell bodies and intact neurites were counted in each well. Data were obtained from three separate experiments repeated in duplicates.

### Sciatic nerve crush

Sciatic nerve crush was performed on anesthetized adult mice (8–12 weeks old) after treated with P-CTX-1 or vehicle, at the level of external rotator muscles, just distal to the sciatic notch^18^. The exposed nerve was crushed with smooth forceps (Fine Science Tools) for 15 seconds. After surgery, overlying muscle and skin were sutured in two layers using 6-0 epineural suture (Ethilon) and allowed to recover on the heated pads^17^,^18^.

### Sensory and motor functional behavioral tests

Three days after sciatic nerve injury, adult mice (n = 10–12 per group) were habituated for three sessions (30 minutes per session) the week before P-CTX-1 (0.26 ng/g) or vehicle treatments. Baseline readings were taken one hour before P-CTX-1 injections. After P-CTX-1 administration, the body weight and behavioral outcomes were monitored in two sessions (morning and evening) with eight hours apart. Sciatic nerve crush was performed two weeks after the first administration of P-CTX-1 in which all the behavioral parameters returned to the baseline values. After injury, sensory (pinprick test) and motor functional recovery (rotarod, sciatic functional index, toe-spreading test, and grip strength of hind limbs and four limbs) were assessed every other day for two months (i.e. 61 days) starting from day 3 after crush injury. We first performed the pinprick sensory assay followed by motor functional assays. All behavioral scoring were done blinded to treatments. Detailed procedures of pinprick sensory assay, rotarod, sciatic functional index (SFI), toe spreading test, and grip strength test were described in Supplementary Methods.

### Electromyography (EMG) recording

Mice were anesthetized with ketamine (100 mg/kg)/xylazine (10 mg/kg), and placed under a heat pad to avoid hypothermia. Site of electrodes were cleaned with ethanol before and after the implantation and returned the animal their respective cages. EMG was recorded (n = 5 per group) 2 months after crush injury (endpoint of behavior studies) using custom made monopolar Teflon-coated electrodes. Recording electrode was inserted subcutaneously into belly of gastrocnemius muscle and reference at Achilles tendon for gastrocnemius muscle EMG recording. For interosseous muscle EMG, recording electrode was inserted into the first muscle and reference electrode into the fourth muscle of the same foot. Sciatic nerve was stimulated proximally (active electrode at the sciatic notch and passive electrode at base of tail) distally (active electrode at Achilles tendon and passive electrode into gastrocnemius muscle. Proximal and distal stimulation was used for compound muscle action potential (CMAP) of interosseous muscle, while only proximal stimulation was used for gastrocnemius muscle. EMG signals were sampled at 5 kHz (Blackrock microsystem, data acquisition set-up, USA), amplified for 1000 times and passed through a bandpass filter with low and high cut-off frequency settings of 10 and 3000 Hz, respectively. The amplified EMG signal was subsequently passed through a low-pass (i.e. 2000 Hz) digital filter to remove aliasing in the 2000–3000 Hz range; any DC offset was also removed at this time. The amplitude of the CMAP was measured from the peak of the negative deflection to the peak of the positive deflection using Spike 2 (Cambridge Electronic Design Limited, England). Mean CMAP amplitude was then calculated accordingly^44^.

### Immunohistochemistry

At the endpoint of behavior studies (2 months after crush injury), mice were perfused with 4% paraformaldehyde (PFA). Sciatic nerve and lateral plantar muscles were dissected, post fixed, cryoprotected and frozen in OCT (Tissue-Tek).

### Axon quantification

PFA-fixed sciatic nerves (n = 3–4 per group) were divided into 5 mm segments from 5-mm proximal to the crush site to the level of the flexor retinaculum in the ankle, 25 mm in total length proximal. After trifurcation, tibial nerves were quantified in the distal segments. Fluorescent images from each 4-μm-thick transverse section of sciatic nerve segment immunostained with anti-neurofilament NF200 antibody (1:2,000, Millipore) were taken. The total number of axons was quantified using ImageJ software as previously described^18^. At least 5–8 random sections were quantified per mouse.

### NMJ analysis

PFA-fixed lateral planter muscle 20-μm-thick cryosection were immunostained with anti-α-bungarotoxin (1:1,000, Molecular Probes) and anti-neurofilament NF200 (1:2,000, Millipore) antibodies. Every fourth section was quantified. Reinnervation was quantified for overlapping NF200 and α-bungarotoxin immunoreactivity, and 3 categories could be identified: innervated - fully innervated; intermediate - partially innervated; denervated - no innervation. NMJ analysis was performed on 6–8 mice per group and at least 600 NMJs were counted per mouse^18^.

### Statistics

Data were presented as mean ± S.E.M. Data were analyzed by Student’s *t*-test (2 groups) and 1-way ANOVA with post-hoc Bonferroni test (>2 groups) where appropriate. Animal behavior data were analyzed by 2-way ANOVA with repeated measures, followed with Bonferroni post-hoc test.

## Additional Information

**How to cite this article**: Au, N. P. B. *et al*. Ciguatoxin reduces regenerative capacity of axotomized peripheral neurons and delays functional recovery in pre-exposed mice after peripheral nerve injury. *Sci. Rep.* **6**, 26809; doi: 10.1038/srep26809 (2016).

## Supplementary Material

Supplementary Information
