# Correction: Ciguatoxin reduces regenerative capacity of axotomized peripheral neurons and delays functional recovery in pre-exposed mice after peripheral nerve injury

**DOI:** 10.1038/s41598-026-67833-y

**Published:** 2026-09-01

**Authors:** Ngan Pan Bennett Au, Gajendra Kumar, Pallavi Asthana, Chung Tin, Yim Ling Mak, Leo Lai Chan, Paul Kwan Sing Lam, Chi Him Eddie Ma

**Affiliations:** 1 Department of Biomedical Sciences, City University of Hong Kong, Tat Chee Avenue, Hong Kong; 2 Department of Mechanical and Biomedical Engineering, City University of Hong Kong, Tat Chee Avenue, Hong Kong; 3 Centre for Biosystems, Neuroscience, and Nanotechnology, City University of Hong Kong, Tat Chee Avenue, Hong Kong; 4 State Key Laboratory in Marine Pollution, City University of Hong Kong, Tat Chee Avenue, Hong Kong; 5 Shenzhen Key Laboratory for the Sustainable Use of Marine Biodiversity, Research Centre for the Oceans and Human Health, City University of Hong Kong Shenzhen Research Institute, Shenzhen, China; 6 Department of Biology and Chemistry, City University of Hong Kong, Tat Chee Avenue, Hong Kong

Correction to: *Scientific Reports* https://doi.org/10.1038/srep26809, published online 27 May 2016

This Article contains errors.

In the Original Article, specifically in Figure 4a part of the image in the “solvent control (SC)” group was erroneously used to represent the “SC + TTX” group. The authors acknowledge the errors and have to the best of their ability reconstructed the original figure using the materials still available. Specifically, the **SC + TTX** has been replaced to correct the error of overlapping in the SC panel. The **CTL** panel has been replaced with a verifiable source image from the same experimental condition, as the original source image could not be recovered. The **25 pg/ml P-CTX-1 + TTX** panel have been replaced with a verifiable source image from the same experimental condition, as the original source image could not be recovered. The errors made do not affect the major conclusions or the quantification of neurite outgrowth in the first place presented in our manuscript. The corrected Figure 4a is shown below:

**Fig. 4:**
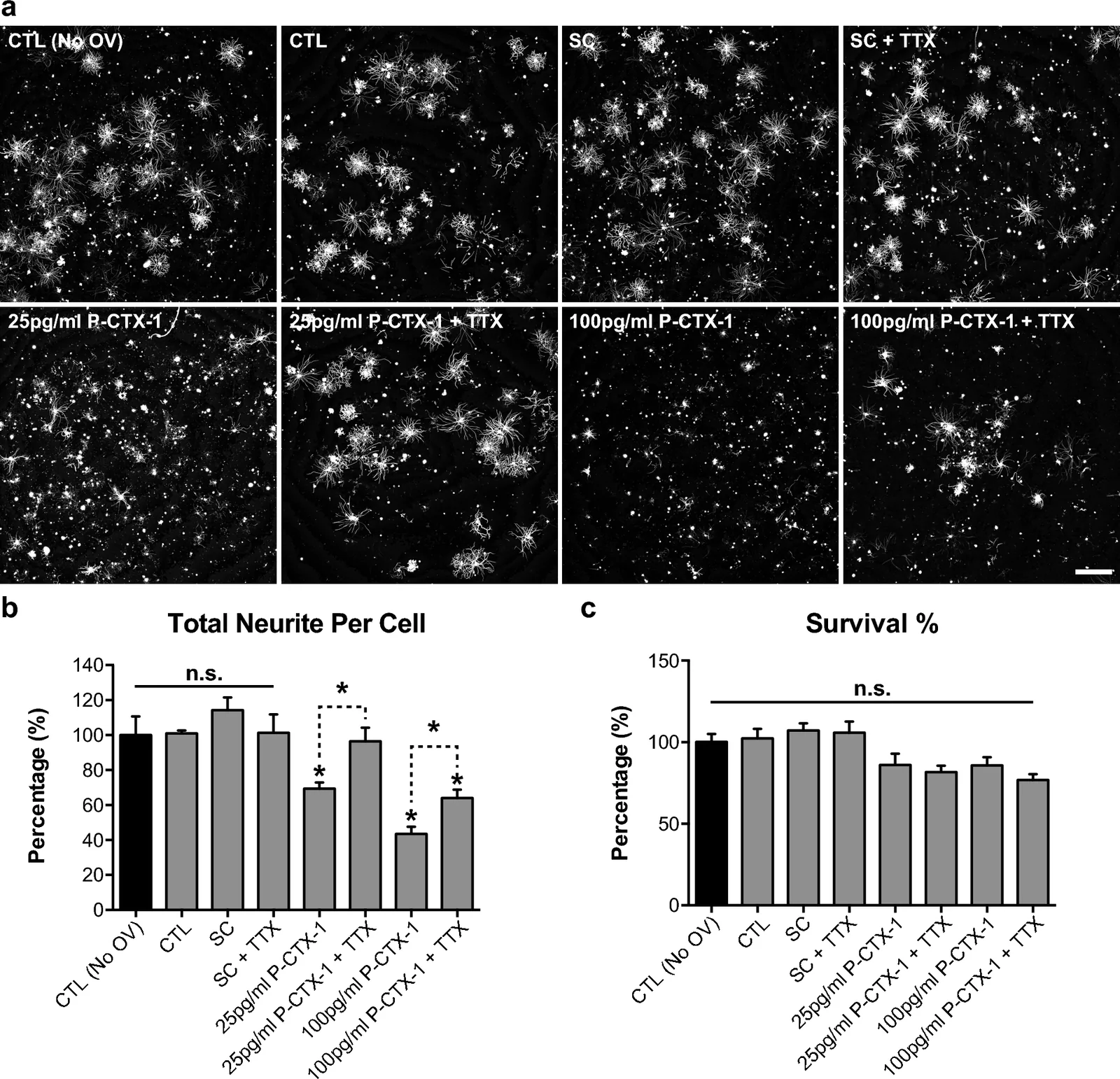
**Blockade of excess sodium ion influx abolishes the growth inhibitory effects of P-CTX-1 on DRG neurons *in vitro***. (**a**) Representative photomicrographs of DRG neurons immunolabelled with antibodies against anti-β -tubulin III. DRG neurons treated with 0.3 μM TTX were able to extend their neurites to lengths comparable to the solvent controls (SC, 0.13% methanol) in the presence of 25 pg/ml P-CTX-1. TTX-treated DRG neurons exhibited significantly longer neurites in the presence of 100 pg/ml P-CTX-1 than those treated with 100 pg/ml P-CTX-1 alone. Scale bar: 500 μm. (**b**) Blockade of sodium influx with TTX significantly enhanced neurite extension in DRG neurons treated with 25 and 100 pg/ml P-CTX-1. (**c**) Neither P-CTX-1 nor TTX affected the survival of DRG neurons at any of the tested concentrations. The values represented the mean ± SEM values of triplicates; ^*^P < 0.05, one-way ANOVA, followed by Bonferroni’s post hoc test. All treatment conditions, except for CTL (medium only), contained 50 μM ouabain (O) and 5 μM veratridine (V). There was no significant difference in neurite outgrowth between DRG neurons cultured with or without O/V. CTL, control; OV, 50 μM ouabain and 5 μM veratridine; TTX, 0.3 μM tetrodotoxin; n.s., not significant.

The conclusions of the Original Article are not affected. The corrected panels confirm the same overall results as originally reported.

