# Supplementary material for: Ciguatoxin reduces regenerative capacity of axotomized peripheral neurons and delays functional recovery in pre-exposed mice after peripheral nerve injury
Source: Sci Rep. 2016 May 27;6:26809. doi: 10.1038/srep26809 (PMC4882531; doi:10.1038/srep26809)
Supplement: Supplementary Information [file srep26809-s1.pdf]

Supplementary Information

Ciguatoxin reduces regenerative capacity of axotomized peripheral neurons and delays functional recovery in pre-exposed mice after peripheral nerve injury

Ngan Pan Bennett Au[1†], Gajendra Kumar[1†], Pallavi Asthana[1], Chung Tin[2,3] , Yim Ling Mak[4,5], Leo Lai Chan[1,4,5], Paul Kwan Sing Lam[4,5,6], Chi Him Eddie Ma*[1,3,4]

[1]Department of Biomedical Sciences, City University of Hong Kong, Tat Chee Avenue, Hong Kong. [2]Department of Mechanical and Biomedical Engineering, City University of Hong Kong, Tat Chee Avenue, Hong Kong. [3]Centre for Biosystems, Neuroscience, and Nanotechnology, City University of Hong Kong, Tat Chee Avenue, Hong Kong. [4]State Key Laboratory in Marine Pollution, City University of Hong Kong, Tat Chee Avenue, Hong Kong. [5]Shenzhen Key Laboratory for the Sustainable Use of Marine Biodiversity, Research Centre for the Oceans and Human Health, City University of Hong Kong Shenzhen Research Institute, Shenzhen, China. [6]Department of Biology and Chemistry, City University of Hong Kong, Tat Chee Avenue, Hong Kong.

[†] These authors contributed equally to this work.

*Correspondence: Dr. Chi Him Eddie Ma

[1]Department of Biomedical Sciences, City University of Hong Kong, Tat Chee Avenue, Hong Kong. [3]Centre for Biosystems, Neuroscience, and Nanotechnology, City University of Hong Kong, Tat Chee Avenue, Hong Kong. [4]State Key Laboratory in Marine Pollution, City University of Hong Kong, Tat Chee Avenue, Hong Kong

Email: eddiema@cityu.edu.hk

Phone: (852)-3442-9328 Fax: (852)-3442-0549

This file includes:

Supplementary Methods and References

Supplementary Figure S1-S4

Supplementary Table S1

Supplementary Methods

Pinprick sensory assay

After a 30-minute habituation period by placing the mice on wire mesh, an Austerlitz insect pin (size 000) (Fine Science Tools) was gently applied to the lateral plantar surface of the hind paw (where the sciatic nerve crush was performed) from heel to toe [1]. The most lateral part of the hind paw was divided into 5 areas. The pin was applied twice from the most lateral toe (score = 5) to heel (score = 1). A brisk withdrawal of hind limb is considered as positive response and the mouse is graded 1 with a maximum score of 5. The mouse scored 0 if it failed to give any responses out of those five areas [1].

Rotarod

Motor coordination was evaluated using rotarod apparatus (LE8500, Bioseb). Mice were trained at initial speed of 4rpm and then progressively increased to accelerated mode for 2 minutes. Mice with 60 seconds retention time on rotarod spindle was included in the experiment. Rotarod was kept in accelerated mode (4-40 rpm) for 5 minutes during the assessment, and the retention time was recorded. Average of five trials were performed and animals were given a minimum of 10 minutes interval between each trial [2].

Sciatic functional index (SFI)

Mice were allowed to walk down a 60cm track after inking their hind paws. Baseline measurements were taken on three separate days. From the footprints, the SFI value using the formula as follows: sciatic functional index = -38.3 (experimental print length - naïve print length)/naïve print length + 109.5 (experimental toe spread - naïve toe spread)/naïve toe spread + 13.3 (experimental intermediary toe spread - naïve intermediary toe spread)/naïve intermediary toe spread - 8.8. Print length was considered as the distance between the third toe to the heel. Toe spread was the distance between the first and the fifth toe. Intermediary

toe spread was the distance between the second and four toe. Four most clear footprints were taken from both 'experimental' (i.e. injured; ipsilateral) and 'naïve' (i.e. uninjured; contralateral) side for SFI measurements. SFI score of 0 was considered as naïve and a set value of -100 for mice unable to plantar place their hind paws mice [3].

Toe spreading test

Motor recovery after crush was assessed using toe spreading motor assay [1]. Mice were gently covered with a thin cotton cloth and the hind paws were exposed for observation. The toe spread reflex were scored as 0 (no spreading of toes); 1 (intermediate spreading of all toes but not sustainable for longer than 2 seconds); or 2 (full spreading of all toes and sustainable for more than 2 seconds). Mice were assessed twice in each behavioral session with intervals of at least 30 minutes.

Grip strength test

Muscular strength was determined quantitatively using grip strength meter (GT-3, Bioseb). To measure four limb grip strength, mice were held by the tail and lowered over the middle of the grid allowing all four paws to grip the metal wire mesh, and gently pulled off. For hind limb grip strength, the forepaws were kept resting on a plastic bar and the hind paws were placed on T bar of grip strength meter, and gently pulled off with constant force until the grip was released. In both measurements, the value at which the mouse left the mesh/bar is designated grip strength (in grams) [4,5]. The mice were tested three times in two different sessions 30 minutes apart. The final value is determined as the average of the five measurements.

References

1. Ma, C. H. *et al.* Accelerating axonal growth promotes motor recovery after peripheral nerve injury in mice. *J Clin Invest* **121**, 4332-4347 (2011).

2. Takemura, Y. *et al.* Brain-derived neurotrophic factor from bone marrow-derived cells promotes post-injury repair of peripheral nerve. *PLoS One* **7**, e44592 (2012).

3. de Medinaceli, L., Freed, W. J. & Wyatt, R. J. An index of the functional condition of rat sciatic nerve based on measurements made from walking tracks. *Exp Neurol* **77**, 634-643 (1982).

4. Meyer, O. A., Tilson, H. A., Byrd, W. C. & Riley, M. T. A method for the routine assessment of fore- and hindlimb grip strength of rats and mice. *Neurobehav Toxicol* **1**, 233-236 (1979).

5. LaMonte, B. H. *et al.* Disruption of dynein/dynactin inhibits axonal transport in motor neurons causing late-onset progressive degeneration. *Neuron* **34**, 715-727 (2002).

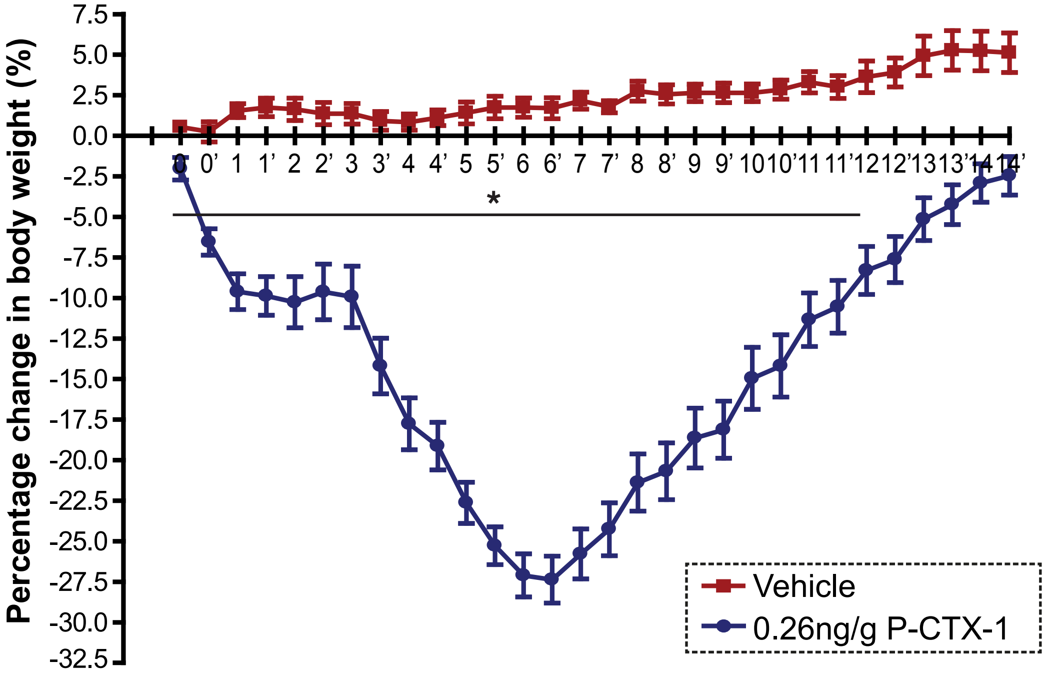

Supplementary Figure S1 | P-CTX-1 exposure induces an acute reduction in body weight. Body weight loss was observed in the P-CTX-1-treated group compared with the vehicle controls, and the weights returned to baseline values within two weeks. (n=10-12 mice per group; mean ± SEM; **P*<0.05, two-way repeated measures ANOVA, followed by Bonferroni's post hoc test).

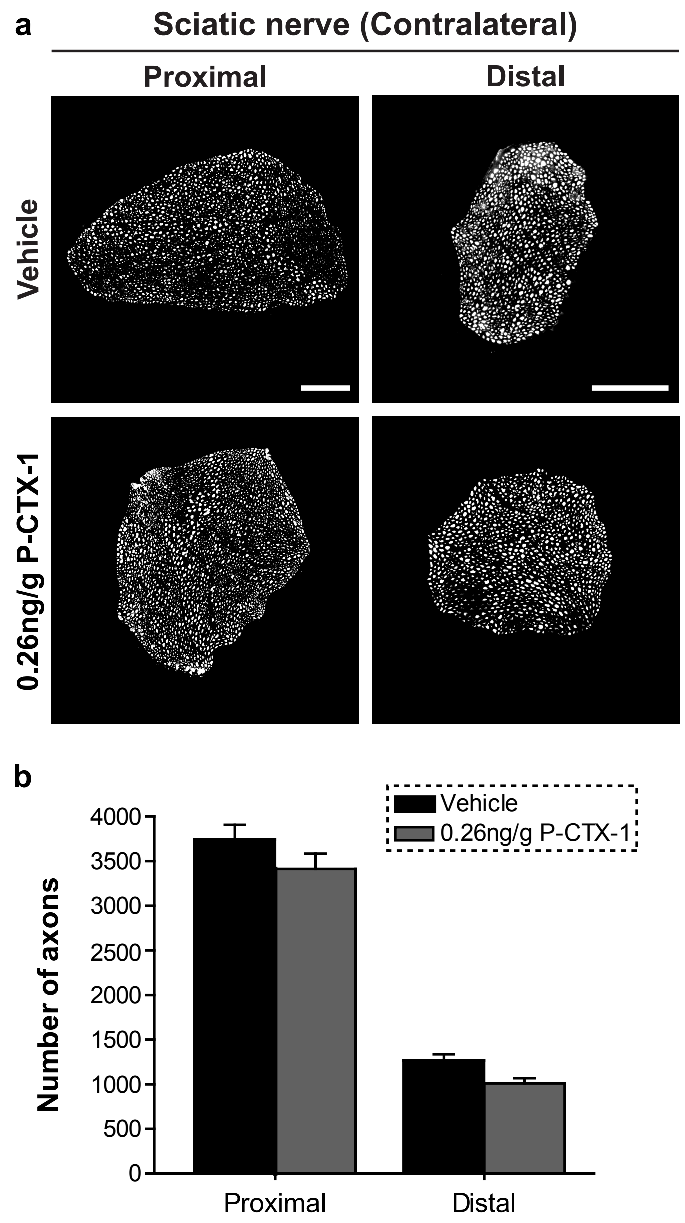

Supplementary Figure S2 | Pre-exposure to P-CTX-1 slightly reduces the number of uninjured axons. (a) Transverse sections (4 μm-thick) of uninjured sciatic nerves were immunostained with an anti-neurofilament (NF200) antibody. Scale bar: 100 μm. **(b)** Quantification of the total number of neurofilament-positive axons indicated a trend towards a reduced total number of axons at the sciatic notch (i.e., proximal) and distal to the sciatic notch. (n=3-4 mice per group and 5-8 sections per mouse; mean ± SEM; **P*<0.05, Student's *t*-test).

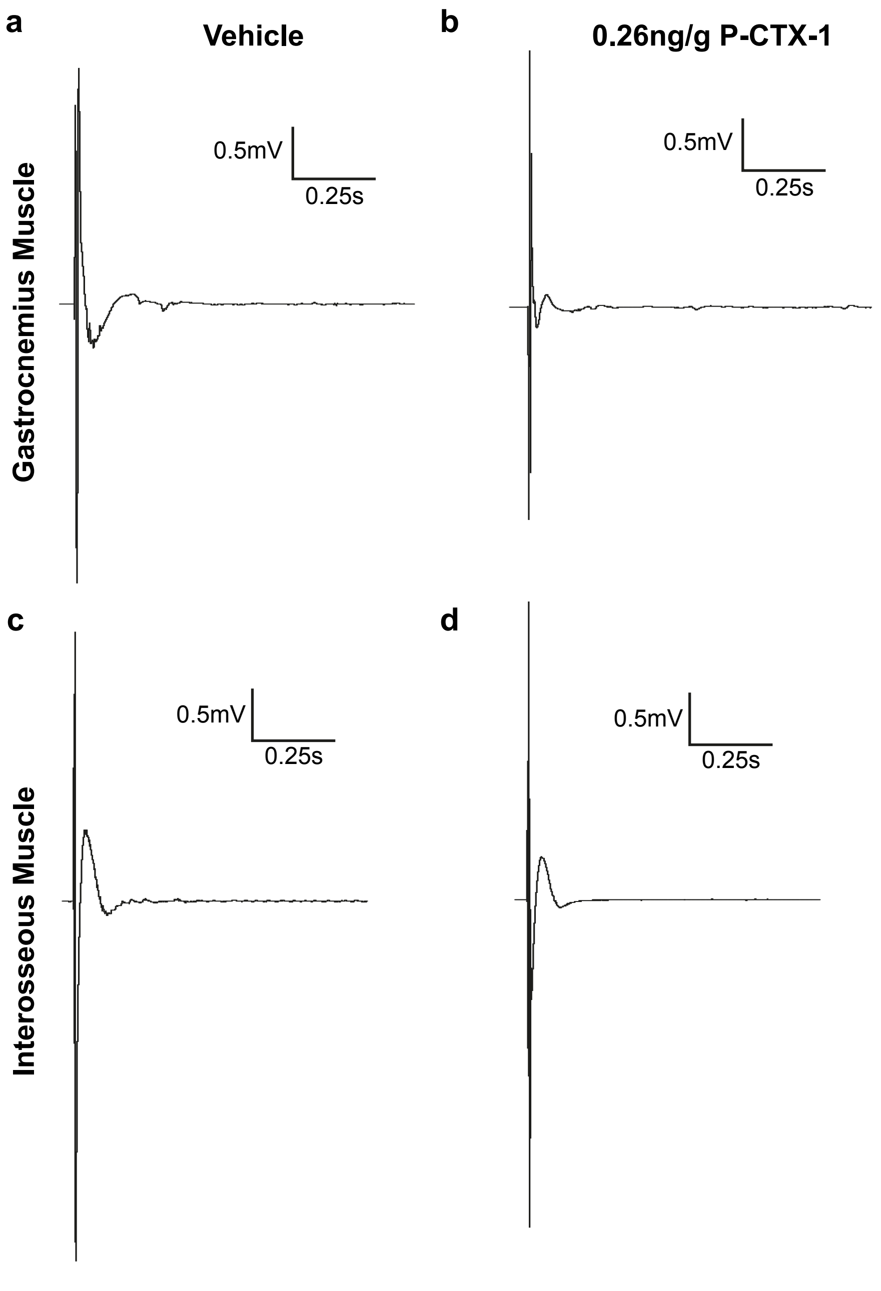

Supplementary Figure S3 | Chronic exposure to P-CTX-1 significantly alters the electrophysiological properties of the gastrocnemius muscle (GCM) and interosseous muscle (IOM) after sciatic nerve crush injury. (a) A representative CMAP trace from the ipsilateral side of a vehicle-treated mouse. Full recovery of the CMAP amplitude was observed in the GCM of the vehicle-treated mice two months after sciatic nerve crush injury. **(b)** The CMAP amplitude of the GCM was significantly decreased in the P-CTX-1-treated mice compared with the vehicle controls two months after sciatic nerve crush injury, indicating motor function impairment, similar to our behavioural data. **(c)** A representative CMAP trace from the ipsilateral side of a vehicle-treated mouse. Full recovery of the CMAP amplitude of the IOM in the vehicle-treated mice was observed two months after sciatic nerve crush injury. **(d)** The CMAP amplitude of the IOM was significantly reduced in the ipsilateral side of the P-CTX-1-treated mice, confirming our NMJ innervation data.

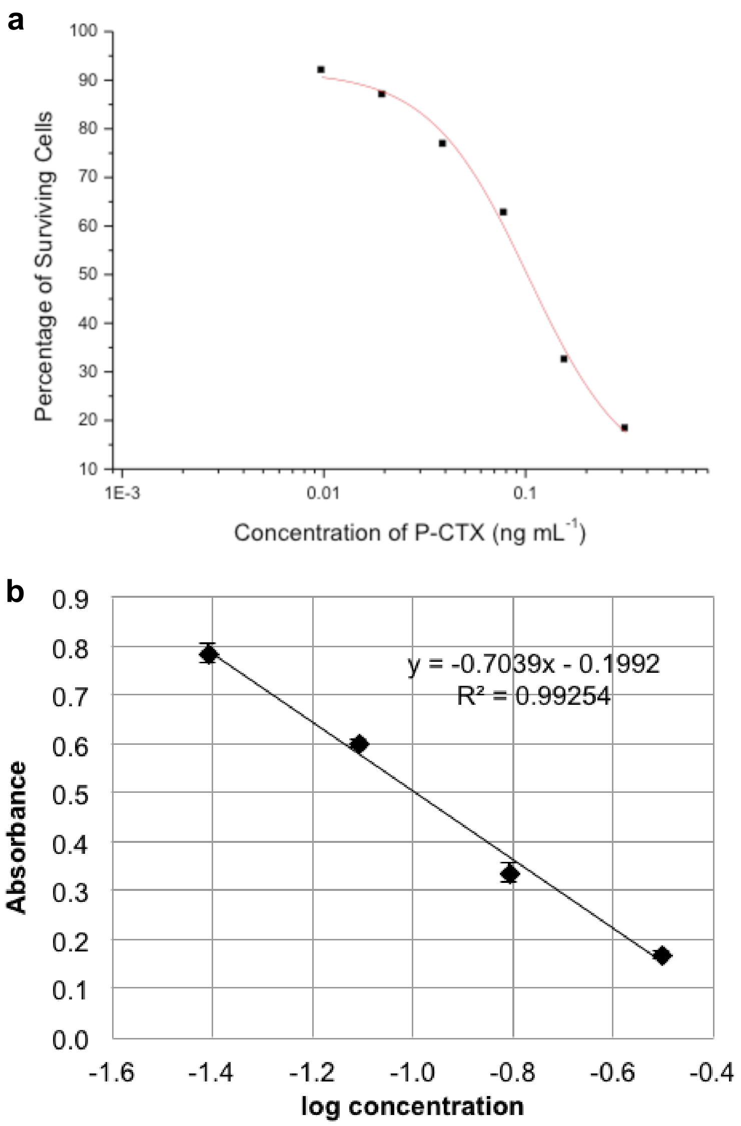

Supplementary Figure S4 | Determination of P-CTX-1 concentration in the mouse tissue samples based on the dose-dependent response of Neuro-2a cells to P-CTX-1. (a) Dose-response curve of the Neuro-2a cells in response to the ciguatoxin (R^2=0.99536), with an LC_{50} of 0.103±0.0173±ng/ml. **(b)** An MNA calibration curve was used to determine the concentrations of P-CTX-1 in the various mouse tissue samples.

	2 hours						
	P-CTX-1 concentration in tissue (ng/g)			average per sample	S.D. per sample	average	S.D.
Sciatic nerve 1	0.96572	0.86427	0.94088	0.92362	0.05288	**1.07399**	**0.65246**
Sciatic nerve 2	0.49311	0.52618	0.51022	0.50984	0.01654		
Sciatic nerve 3	1.91852	1.60756	1.83946	1.78851	0.16162		
Leg muscle 1	0.32594	0.25331	0.30142	0.29355	0.03695	**0.30761**	**0.07819**
Leg muscle 2	0.18564	0.27721	0.24935	0.23740	0.04694		
Leg muscle 3	0.27784	0.65055	0.24723	0.39187	0.22454		
Liver 1	0.72720	0.73026	0.71964	0.72570	0.00547	**0.69306**	**0.15643**
Liver 2	0.87455	0.88337	0.73383	0.83058	0.08391		
Liver 3	0.56274	0.53679	0.46912	0.52288	0.04833		
Heart 1	0.55881	0.60351	0.52658	0.56297	0.03863	**0.62454**	**0.05509**
Heart 2	0.60195	0.67903	0.64350	0.64149	0.03858		
Heart 3	0.67301	0.61942	0.71509	0.66917	0.04795		
Kidney 1	1.09863	0.64844	0.99406	0.91371	0.23561	**0.75923**	**0.36128**
Kidney 2	1.15934	1.01142	0.88199	1.01758	0.13878		
Kidney 3	0.38277	0.40480	0.25162	0.34640	0.08282		
Stomach 1	0.49704	0.54895	0.43178	0.49259	0.05871	**0.70729**	**0.36166**
Stomach 2	0.52127	0.51612	0.47591	0.50443	0.02483		
Stomach 3	1.31479	1.14042	0.91932	1.12484	0.19819		
Intestine 1	1.06052	1.22037	1.14070	1.14053	0.07993	**0.93741**	**0.21328**
Intestine 2	0.96955	1.00261	0.89724	0.95647	0.05389		
Intestine 3	0.60734	0.74642	0.79196	0.71524	0.09618		
	2 months						
	P-CTX-1 concentration in tissue (ng/g)			average per sample	S.D. per sample	average	S.D.
Sciatic nerve 1	0.14082	0.13105	0.15349	0.14179	0.01125	**0.12790**	**0.01964**
Sciatic nerve 2	0.08427	0.10935	0.14844	0.11402	0.03234		
Sciatic nerve 3	0.14878	0.25934	0.30273	0.23695	0.07938		
Leg muscle 1	0.00102	0.00117	0.00085	0.00101	0.00016	**0.00079**	**0.00034**
Leg muscle 2	0.00110	0.00091	0.00083	0.00095	0.00014		
Leg muscle 3	0.00040	0.00041	0.00037	0.00039	0.00002		
Liver 1	0.03492	0.02599	0.02674	0.02922	0.00496	**0.02833**	**0.00125**
Liver 2	0.03244	0.02569	0.02423	0.02745	0.00438		
Liver 3	0.02699	0.02123	0.01765	0.02196	0.00471		
Heart 1	0.02598	0.02993	0.03095	0.02895	0.00262	**0.01944**	**0.01346**
Heart 2	0.00918	0.01018	0.01041	0.00992	0.00065		
Heart 3	0.02841	0.03811	0.04776	0.03810	0.00968		
Kidney 1	<LOQ	<LOQ	<LOQ	<LOQ	N.A.		
Kidney 2	<LOQ	<LOQ	<LOQ	<LOQ	N.A.		
Kidney 3	<LOQ	<LOQ	<LOQ	<LOQ	N.A.		
Stomach 1	0.09316	0.06691	0.09650	0.08552	0.01620	**0.08407**	**0.00205**
Stomach 2	0.08795	0.07494	0.08497	0.08262	0.00681		
Stomach 3	0.09850	0.17734	0.11491	0.13025	0.04160		
Intestine 1	0.01458	0.01090	0.01276	0.01275	0.00184	**0.01325**	**0.00071**
Intestine 2	0.01444	0.01229	0.01455	0.01376	0.00127		
Intestine 3	0.01431	0.01665	0.02516	0.01871	0.00571		

Supplementary Table S1 | Concentration of P-CTX-1 in mouse tissue samples after P-CTX-1 exposure. The concentration of P-CTX-1 in individual tissue samples was determined based on the standard curve as shown in Supplementary Figure S6. LOQ, limit of quantification; N.A., not available.
